# Diversity of the Chemical Profile and Biological Activity of *Capsicum annuum* L. Extracts in Relation to Their Lipophilicity

**DOI:** 10.3390/molecules26175215

**Published:** 2021-08-27

**Authors:** Barbara Chilczuk, Beata Marciniak, Renata Kontek, Małgorzata Materska

**Affiliations:** 1 Group of Phytochemistry, Department of Chemistry, Faculty of Food Science and Biotechnology, University of Life Sciences in Lublin, Akademicka 15, 20-950 Lublin, Poland; barbara.chilczuk@up.lublin.pl; 2 Department of Molecular Biotechnology and Genetics, Faculty of Biology and Environmental Protection, University of Lodz, Banacha 12/16, 90-237 Lodz, Poland; beata.marciniak@biol.uni.lodz.pl (B.M.); renata.kontek@biol.uni.lodz.pl (R.K.)

**Keywords:** *Capsicum annuum*, phenolic compounds, antiradical activity, anticancer properties, LC-QTOF-MS

## Abstract

Ethanol extracts of two types of pepper (sweet and hot) were separated into fractions with increasing lipophilicity. After drying the extracts and fractions, their chemical composition, anti-radical activity in the DPPH radical system, and cytotoxic activity against PC-3 and HTC-116 cells were determined. A detailed qualitative analysis of the fractions was performed with the LC-QTOF-MS method. It was found that the chemical composition of pepper fractions did not always reflect their biological activity. The highest antiradical activity was detected in the fraction eluted with 40% methanol from sweet pepper. The highest total content of phenolic compounds was found in an analogous fraction from hot pepper, and this fraction showed the strongest cytotoxic effect on the PC-3 tumour line. The LC-MS analysis identified 53 compounds, six of which were present only in sweet pepper and four only in hot pepper. The unique chemical composition of the extracts was found to modulate their biological activity, which can only be verified experimentally.

## 1. Introduction

A number of compounds synthesised by plants as a result of secondary metabolism exhibit many interesting biological properties [1]. This is related to the fact that they fulfill specific physiological functions in plants, e.g., they help protect cells against infections [2], have pigmentation properties [3], and protect plant cells against too strong radiation [4]. This group includes phenolic acids, flavonoids, alkaloids, vitamins, etc. [5,6]. The concentration and composition of secondary metabolites depend on the plant species and on external factors, such as climatic conditions, water and temperature stress, or infections [7]. For this reason, diversity of active compounds present in plants determine the chemical and biological activity of the plant extracts [8]. The advantage of natural plant extracts over synthetic biologically active compounds lies in their unique chemical composition, which depends not only on the plant species and growth conditions, but also on the method of preparation and storage of the extracts [9,10]. Therefore, it is not possible to obtain a synthetic extract with the same composition and properties as the original plant. The research conducted in the last decade has largely focused on the search for compositions in plant raw materials that can meet the expectations with regard to their use as a substitute for synthetic food additives, as components of food products with reduced content of synthetic preservatives, or in medicine for prevention of civilization diseases and supporting the treatment of serious diseases [8,11,12]. In their investigations, scientists often use plants that have been known for centuries and are still used in folk medicine. Some of the forgotten species are being rediscovered [13,14].

The plant or substance considered as a potential source of biologically active compounds is first analysed in terms of antioxidant properties [8]. This activity, determined with in vitro methods, may be based on direct and indirect mechanisms of antioxidant activity [8]. In the case of single compounds, many algorithms have been developed that help in the theoretical determination of the activity spectrum. Based on the QSAR analysis, the physical and chemical properties of compounds are calculated and their bioavailability and biological activity are estimated [15,16]. It has been found that lipophilicity is one of the most important physicochemical properties that influences the biological activity of compounds [17]. Structure-activity studies play a key role in the early stages of drug development [17,18]. In the case of plant extracts, which constitute a multi-component matrix, such analyses are useless. Our previous research and the analysis of the available world literature indicate that, in the case of multi-component systems as plant extracts, the antiradical activity does not always reflect their biological activity. Moreover, the biological activity of these systems may manifest itself to varying degrees depending on the reaction medium [19]. This is related to the specific chemical composition of extracts and possible interactions between the contained compounds. For this reason, empirical research is indispensable in such considerations.

Pepper fruits are a valuable source of extracts with strong biological properties expressed as anti-cancer properties [19,20]. The annual pepper (*Capsicum annuum* L.) is a popular vegetable plant widely grown and consumed all over the world. It is a key component of the Mediterranean diet, which is believed to have a beneficial effect by reducing the risk of cardiovascular events [21,22]. Sweet pepper fruits are well known as an excellent source of bioactive compounds, including ascorbic acid (vitamin C), carotenoids (provitamin A), tocopherols (vitamin E), and flavonoids or diterpene derivatives [19]. Spicy varieties additionally contain capsaicinoids, i.e., unique alkaloids whose concentration influences the hotness of fruits. Many of these compounds exhibit diverse biological activities and protect cells from oxidative damage as a result of interactions with oxygen molecules and neutralisation of peroxide radicals [23,24]. This activity is reflected in health-promoting properties of this vegetable present in the daily diet [25].

As shown by the results of our previous research [19], sweet pepper extracts are characterised by anti-carcinogenic activity against prostate cancer cell lines (PC3). However, in the current literature on the biological activity of *Capsicum annuum* extracts, we found no evidence concerning the difference in their qualitative composition between sweet and hot pepper extracts.

The aim of the research was to estimate the relationship between the components of the extracts determined with the LC-QTOF-MS method and the antiradical activity of fractions with variable lipophilicity obtained from ethanol extracts of sweet and hot pepper. Their biological activity was also assessed in terms of anti-cancer properties against human colon cancer HCT116 and prostate adenoma PC3 cell lines.

## 2. Results and Discussion

The active compounds from the pericarp of sweet and hot peppers were extracted with an 80% solution of ethanol in water, as it is the most effective solvent for active compounds [26]. However, due to its versatility in the extraction of active compounds, this solution is characterised by low selectivity. Our previous research showed that ethanol extracts contained both typically hydrophilic (including vitamin C) and lipophilic (vitamin E) compounds and a number of compounds with intermediate lipophilicity, mainly from the group of polyphenols [27]. The extraction efficiency was about 7–8% (Table 1), and the cause of the differences between the sweet and the hot varieties was mainly the dry matter content, which was 7.2 ± 0.19% and 9.08 ± 0.43% in the sweet and hot varieties, respectively. The obtained ethanol extract was subjected to further separation for two reasons: Firstly, to obtain less complex fractions, which allowed a more precise analysis of their chemical composition. Secondly, after drying, the fractions contained a condensed amount of active compounds in a smaller volume, which is particularly desirable in their use as active food additives. As can be seen from the obtained extraction yields, after separating the ethanol extract into three fractions, the highest yield was found for the water fraction—F1 (Table 1). This was not surprising, as the pepper extract contains a significant amount of sugars, the weight of which was reflected in the weight of the F1 fraction [28]. The next stage of the research was to evaluate the chemical composition and anti-radical activity of the dried fractions and the initial extract of two pepper varieties. The results of the tests are summarised in Table 1. Many methods are available for determining the antioxidant activity, but the DPPH radical assay seems to be the most commonly used and was applied in these investigations [6,29,30]. Among the analysed samples, the fraction with intermediate lipophilicity (F2) was characterised by the highest content of active ingredients: Total phenolic (TP), total flavonoid (TF), and total dihydoxycinnamic acids (TDHCA), which was manifested in the highest anti-radical activity (AA). Moreover, other studies have suggested that TP and AA are closely related to each other [31,32]. The comparison of the composition of the analysed fractions from sweet and hot peppers revealed significant differences between them. Although the highest concentration of phenolic compounds was found in the F2 fraction in both cultivars, their content in the hot pepper fraction was almost 30% higher than in the analogous fraction from sweet pepper. In turn, sweet pepper contained over 20% more phenolic compounds in the F1 fraction than hot pepper. However, this was not reflected by the anti-radical properties of this fraction. The F3 fractions of both tested pepper cultivars were the most similar in terms of the sum of phenolic compounds. The observed differences in TP, TF, and TDHCA in the sweet and hot pepper varieties indicate a different course of secondary metabolism of their components, which modulates their unique chemical composition. This is obvious, since hot peppers synthesise capsaicinoids at the expense of phenolic compounds in the phenylpropanoid metabolism pathway [33,34]. This may be the cause of the lower TP content in the F1 fraction in the hot cultivar.

In order to determine whether the chemical composition and anti-radical activity reflect the biological activity, the pepper fractions were subjected to the assessment of anti-cancer properties. For this purpose, the HCT116 human colorectal cancer and PC3 prostate adenoma cell lines were used. The L929 line was used as a model of normal cells, i.e., murine fibroblasts from subcutaneous adipose tissue, which is recommended for research of the cytotoxicity of chemical compounds and with which the results obtained were compared [14,35]. Murine L929 fibroblasts were used in the cytotoxicity analyses of the obtained extracts for technical reasons, since the preparation of the suspension of these cells is much faster and requires incubation for 24 h, while the preparation of the suspension of cells from human fibroblasts requires 72 h [36]. It was shown that the effect of the extracts and fractions from the pericarp of both peppers depended on the type of neoplastic cells. The prostate tumour cell line (PC-3) was the most sensitive to the effect of the pepper extract and the fraction with variable lipophilicity (Table 2). A significantly lower degree of cytotoxicity was noted in the experimental series with the use of normal cells—L929. In the PC-3 neoplastic cells, the strongest cytotoxic effect was exerted by the F3 fraction from sweet pepper and F2 from hot pepper. The comparison of the cytotoxic properties of the extracts and fractions of both pepper cultivars showed clearly more favourable anticancer properties of the hot pepper extracts, where both the ethanol extract and its fractions showed stronger cytotoxic properties than the analogous fractions of sweet cultivar. Only the F3 fraction from sweet pepper was characterised by an almost two-fold higher cytotoxic activity than the F3 fraction from hot pepper.

Since cancer is one of the most common causes of death in the world, efforts of many scientists are focused on finding effective ways to prevent and treat the disease. The cytotoxic properties of chemical compounds and plant extracts are the focus of investigations in many research centers in the world. Due to the variety of neoplastic cells and methods for their destruction, the amount of global research on this issue is increasing. There are a few examples of investigations of compounds derived from the *Capsicum annuum* L. Hot pepper pericarp extracts and pure capsaicin were analysed for their cytotoxic effect on B104 neuroblastoma cells [20]. The authors of the study demonstrated the anti-tumour effect of capsaicin on the neoplastic line. However, they did not find this activity in ethanol extracts from peppers, which contained, e.g., vitamins E and C and quercetin in addition to capsaicin at a concentration of 0.5–0.2 mmol/L. The authors proposed that co-extracted compounds within the ethanolic extract interact antagonistically with the cytotoxic effect of capsaicin. On the other hand, a stronger cytotoxic effect of multi-component pepper extracts than that of pure capsaicin has been confirmed in studies on many human tumour cell lines: Human hepatoma (HepG2), human gastric adenocarcinoma (AGS), human breast cancer (MCF-7), human cervical carcinoma (HeLa), human colon carcinoma (HT-29), and human lung carcinoma (A549) [37,38,39]. The cytotoxic activity of plant extracts differs between cell lines [19,39]. This is related to the different levels of sensitivity of cancer cells to the extracts, as demonstrated in the present study in two cell lines, of which only one (PC-3) was sensitive to the applied factors. In the case of the HTC116 line, none of the tested fractions showed the desired cytotoxic activity (Table 2). Comparing the sweet and hot pepper variety, a stronger antitumor activity against the PC-3 line was observed for the hot pepper extracts (Table 2). However, the fraction from hot pepper, where capsaicin occurred (F3) was less active than the corresponding fraction from sweet pepper. On the one hand, these results indicate a possible synergistic effect of the components of the analysed fractions, especially in the case of the F3 fraction from sweet pepper. On the other hand, an inhibitory effect of the components on their biological activity cannot be ruled out, which was found in the F2 fraction of the same pepper variety. 

The qualitative analysis of the extracts of both pepper varieties and their fractions was performed with the LC-QTOF-MS method (Table 3). Based on the mass spectra of the existing patterns [19,33,40] and the literature data on the LC-MS analysis of pepper extracts [41,42], 53 compounds were identified in the tested fractions. The presence of sugars (glucose and sucrose) was confirmed in the ethanol extracts of both pepper varieties and in fractions F1 and F2, which confirms the thesis about their influence on the extraction efficiency. Most of the identified compounds were present in both sweet and hot pepper extracts. However, the presence of some compounds was confirmed in only one of them. In the case of sweet peppers, these were gallic acid (Rt = 0.448 min), capsianoside VII (Rt = 0.905 min), gentisic/protocatechuic acid (Rt = 4.325 min), capsiate (Rt = 7.308 min), methyl cinnamate (Rt = 10.618 min), and capsidiol (Rt = 13,342 min). In turn, the presence of luteolin (Rt = 0.631 min), capsaicin (Rt = 7.807 min), dihydrocapsaicin (Rt = 8.690 min), and capsianoside I methyl (Rt = 8.988 min) was confirmed only in the hot pepper. The fractionation of the ethanol extract allowed separation of some compounds present in the initial ethanol extract. However, not all compounds were separated into fractions. For this reason, each of the fractions had a different qualitative (Table 3) and quantitative composition, which was confirmed by the spectrophotometric analyses (Table 1). This diversity also modulated the chemical activity marked as anti-radical properties (Table 1) and anti-cancer activity (Table 2). Both ethanol extracts from pepper and their fractions constitute a multi-component composition of active compounds [33,40,43]. Variable concentrations and interactions of these compounds determine their physicochemical properties and biological activity.

The possibility of interactions between fraction components should be explained by the presence of hydrophilic compounds in more lipophilic fractions and *vice versa*. For example, succinic, cinnamic, and chlorogenic acids were present only in the F1 fraction, the presence of sinapic acid was recorded in fraction F2, while *p*-coumaric acid in fraction F2 and sometimes in F3. Additionally, the same compounds eluted differently depending on the matrix composition (sweet and hot peppers), such as malic, quinic, citric, and ferulic acids (Table 3).

## 3. Materials and Methods

### 3.1. Plant Material and Procedure of Extraction and Fractionation

The research material consisted of the fruits of two varieties of pepper: Ajfos—sweet (PS) and Capel Hot—hot (PH). Fruits in the full maturity stage selected for the analysis were washed, dried, and cut into 1 cm^3^ cubes after removal of seeds.

Weighed pepper samples (50 g) were homogenised in 80% aqueous ethanol (1 L) for 15 min using a Diax 500 homogeniser. (Athena DiaX GmbH, Barlin, Germany) The extract was filtered on a vacuum pump and then its volume was reduced to 1 mL on a Buchi vacuum evaporator at 40 °C (BÜCHI Labortechnik AG, Flawil, Switzerland). In order to separate the compounds into groups with a similar nature and properties, the concentrated extract was divided into three fractions with varied hydrophilicity with the solid phase extraction (SPE) method. The division was carried out on columns SPE-C18. The hydrophilic fractions were eluted with water (F1), the intermediate lipophilic fraction was extracted with 40% methanol in water (F2), and the group of compounds with the highest hydrophobicity was eluted with 70% methanol in water (F3). The volume of the fractions obtained was reduced on a Buchi vacuum evaporator at 40 °C, frozen, and freeze-dried. Freeze drying was carried out for 72 h using a freeze dryer (Free Zone 12 lyophiliser, Labconco Corporation, Kansas City, MO, USA) at −80 °C and 0.04 mbar. The freeze-dried samples were stored in airtight containers protected from light until analysis.

### 3.2. Dry Mass Assessment

The dry weight of the extracts was determined on the basis of the weight of its lyophilisate. After the freeze-drying process, the extracts were weighed to determine the extraction yield, which was expressed as mg/100 g of fresh raw material.

### 3.3. Chemical Analysis

Before the analysis, the dried extracts were dissolved in a water or methanol-water (1:1) solution to obtain a starting solution with a concentration of 1 mg/mL. The starting solution was diluted before the analysis, if necessary.

#### 3.3.1. Total Phenolic Compounds (TP)

The total content of phenolic compounds was determined with the Folin-Ciocalteu reagent method [29,44]. The reaction mixture consisted of the following reagents: 60 µL of the tested extract, 1.5 mL of Folin’s reagent diluted in water in a ratio of 1:10, 1.2 mL of sodium bicarbonate (75 g/L), and 0.54 mL of distilled water. The reaction mixture was stored at room temperature for 30 min. Absorbance was measured at a wavelength of λ = 760 nm. The sum of phenolic compounds was expressed as chlorogenic acid equivalents [mg chlorogenic acid/g lyophilised extract].

#### 3.3.2. Total Flavonoids (TF)

The sum of flavonoids contained in the analysed extracts was determined with the AlCl_3_ method [45]. The reaction mixture consisted of the following reagents: 0.5 mL of the tested extract, 1.5 mL of ethanol 96%, 0.1 mL of AlCl_3_ (10%), 0.1 mL of sodium acetate (1 M), and 2.8 mL of distilled water. The reaction mixture was stored at room temperature for 40 min and the absorbance was measured at a wavelength λ = 415 nm. The sum of flavonoids was expressed as quercetin equivalents [mg quercetin/g lyophilised extract].

#### 3.3.3. Total dihydroxycinnamic acids (TDHCA)

The content of dihydroxycinnamic acids was determined in Arnov’s method [46]. The reaction mixture consisted of the following reagents; 0.5 mL of the tested extract, 1 mL of HCl (0.5 M), 1 mL of Arnov’s reagent (10 g NaNO_2_ + 10 g Na_2_MoO_4_ in 100 mL of distilled water), and 1 mL of NaOH (2.125 mol/L). The absorbance was measured after 20 min at a wavelength λ = 525 nm. The TDHCA was expressed as chlorogenic acid equivalents [mg chlorogenic acid/g lyophilised extract].

### 3.4. Biological Activity

#### 3.4.1. Antiradical Activity (AA)

The antiradical activity was determined in the system with the 1,1-diphenyl-2-picrylhydrazyl radical (DPPH•) according to the method proposed by Conforti et al. [47]. The reaction mixture consisted of the following reagents: 100 µL of extract and 4 mL of a 0.1 mM DPPH solution. The sample was stored at room temperature for 30 min, and then the absorbance was measured at λ = 515 nm. For quantitative comparisons, the antiradical activity was expressed as Trolox equivalent on the basis of a standard curve prepared for this compound. The results were expressed as μmol of Trolox/g of dry extract.

#### 3.4.2. Anticancer Activity

The cytotoxicity of the variable lipophilicity subfractions was assessed on two tumour cell lines: The human prostate cancer (PC-3) and the colon cancer (HTC116). Cells of the PC-3 line carry homozygous mutations in the PTEN and TP53 genes. The cytotoxicity of the fractions was assessed on the mouse L929 fibroblast cell line used as a reference for normal cells. The in vitro cytotoxicity tests were carried out in accordance with the PN-EN ISO 10993-5: 2009 standard. The HCT116, PC-3, and L929 cell lines were obtained from the American Type Culture Collection (ATCC, Rockville, MD, USA). The PC-3 cell line was grown in a DMEM-F12 medium, while the HCT116 cells were kept in a RPMI-1640 medium. Normal cells were grown in a IMDM medium.

All the media used were supplemented with foetal bovine serum (10%) and 1% penicillin/streptomycin. The cells were grown in an incubator at a constant temperature of 37 ℃ and CO_2_ content of 5%. Regular 2-week passages were performed using 0.025% trypsin/EDTA after the cells had reached 90% confluence. The PC-3 and HTC116 cell lines were screened for mycoplasma contamination. MTT is a quantitative colorimetric method used to determine the state of cellular metabolism after the treatment with the tested compounds. It is used to assess the cytotoxic effects of chemicals on various cell types [48]. MTT [3-(4,5-dimethyl-2-thiazolyl)-2,5-diphenyl-2H-tetrazolium bromide] is soluble and, upon dissolution, transforms into an insoluble formazan. This product is impermeable to cell membranes and therefore accumulates in metabolically active cells. The assay was optimised for the cell lines and chemicals used in the experiments. Cancer cells and mouse fibroblasts (L929) were cultured for 24 h (37 °C, 5% CO_2_) in 96-well microplates at a density of 6–8 × 103 cells/well. The cells were then incubated with the freshly prepared tested extracts for another 72 h in the same conditions.

At the end of the incubation, an MTT solution (5 mg/mL) was added for another 4 h. The contents of the wells were dissolved by adding 100 µL of DMSO. Absorbance was measured spectrophotometrically using a microplate reader (BioTek Instruments, Inc., Winooski, VT, USA) at 570 nm using DMSO as a blank. The results were analysed in GraphPad Prism software version 7.0 (GraphPad Prism Software Inc., San Diego, CA, USA) and presented as IC50 values. 5-Fluorouracil (a pyrimidine analogue used as an anti-cancer agent in the treatment of solid tumours, including colon, rectal, breast, stomach, pancreas, ovary, bladder, and liver cancer) was used as a positive control. Results are presented as the mean ± SEM of the replicates [49,50].

### 3.5. LC-MS Analysis

The qualitative analysis of the obtained pepper extracts and the isolated fractions was carried out using the LC-QTOF-MS method. The analyses were performed using an Agilent Technologies 1290 series liquid chromatograph coupled to an Agilent Technologies 6530 Q-TOF LC/MS high-resolution mass spectrometer (Agilient Technologies, Palo Alto, CA, USA). The chromatographic separation was carried out on a C18 column, 2.1 × 10 mm, grain 1.8 mm. The mobile phase consisted of 0.1% acetic acid in acetonitrile (A) and water (B) in a solvent gradient ratio in which the concentration of solvent A was as follows: 0–5 min: 20–45%; 5–10 min: 45%; 10–15 min: 95%; at 15–16 min of the program, the eluent composition returned to the initial gradient. The column was equilibrated for 3 min before the next injection. The mobile phase flow was 0.4 mL/min and the volume of the dosed sample was 5 µL. Mass spectra were obtained in the mass range 100–2000 Da with a scan time of 1.0 s operated in positive (ESI +) and negative (ESI-) ionisation modes. Parameters: Capillary voltage 3500 V, nitrogen gas temperature 300 °C at a flow rate of 5 L/min, shield gas temperature 300 °C at a flow rate of 8 L/min, and a nebulizer pressure of 35 psi. Data were collected using the “MassHunter Acquisition” and MassHunter Qualitative Analysis” software (Agilent Technologies, Inc. Santa Clara, CA, USA). The system “Personal Compound Database (PCD) and Library Software” was used to interrogate the database and library directly, where the identification of the compound was carried out using the Find Compound by Formula (FBF) algorithm. The identification of compounds in this algorithm is based on the analysis of isotope patterns (the pattern can be used as a filter for elemental composition assignment). In the case of matching the isotope pattern, the deviation (ppm) between the isotopes and the monoisotopic mass, among others, is taken into account. Contributions to the overall score were set as follows: Mass score 100, isotope abundance score 60, isotope spacing score 50, retention time 100.

### 3.6. Statistical Analysis

The results were performed in three replications, and the data were expressed as the mean ± SD. The significance of differences between the means was determined with the LSD one-way ANOVA test with 5% error probability. The dose-response curve was obtained by plotting the percentage of inhibition versus the concentration. The inhibitory concentration of 50% (IC50) was calculated by linear and nonlinear regression analyses. Statistical comparisons were performed with the help of the Statgraphics Centurion software, version XVI (Statgraphics Technologies Inc. The Plains, VA, USA).

## 4. Conclusions

The obtained results confirmed the thesis that the chemical composition and antiradical activity of multi-component plant extracts and their fractions are not always related to their biological activity. Moreover, the cytotoxic activity of the tested fractions cannot be ruled out based on the lack of anti-radical activity. This was demonstrated for the F1 fraction from hot pepper (Table 1 and Table 2) and the PC-3 tumour line. In the case of multi-component systems, such as plant extracts and their fractions, experimental analyses are indispensable, which can confirm or exclude their activity in relation to the selected medium.

## Figures and Tables

**Table 1:** Extraction yield, phenolic content, and antioxidant activity of ethanolic extract (E) and water (F1), methanol-water 40% (F2), and 70% (F3) fractions obtained from sweet (PS) and hot (PH) pepper fruit.

		Yield ^2^	TP ^3^	TF ^4^	TDHCA ^5^	AA ^6^
PS ^1^	Extract	6.98 ^a^ ± 0.05	17.16 ^d^± 0.19	7.56 ^e^ ± 0.3	27.05 ^e^ ± 0.08	85.11 ^b,c^ ± 0.89
	F1	5.82 ^b^ ± 0.07	22.53 ^c^± 0.55	7.32 ^e^ ± 0.01	26.08 ^e^ ± 0.13	79.78 ^b,c^ ± 0.33
	F2	0.06 ^c,d^ ± 0.01	40.20 ^b^± 0.17	41.59 ^b^ ± 0.5	44.92 ^b^ ± 1.19	394.71 ^a^ ± 5.36
	F3	0.11 ^c^ ± 0.03	23.75 ^c^± 0.06	6.49 ^e^ ± 0.3	33.65 ^c^ ± 0.04	41.11 ^d^ ± 0.83
PH	Extract	7.91 ^a^ ± 0.03	18.14 ^d^ ± 0.18	8.94 ^d^ ± 0.06	30.75 ^d^ ± 0.35	112.20 ^b^ ± 2.69
	F1	6.64 ^a,b^ ± 0.04	17.53 ^d^ ± 0.30	5.40 ^f^ ± 0.01	25.02 ^e^ ± 0.09	98.45 ^b^ ± 0.33
	F2	0.11 ^c^ ± 0.01	51.72 ^a^ ± 1.34	69.45 ^a^ ± 0.56	55.75 ^a^ ± 0.18	376.08 ^a^ ± 7.20
	F3	0.20 ^c^ ± 0.02	22.77 ^c^ ± 0.21	12.15 ^c^ ± 0.15	33.48 ^c^ ± 0.62	57.91 ^d^ ± 1.44

^1^ Results for sweet pepper (PS) quoted from Chilczuk et al. 2020. The values are expressed as the mean ± SD from three independent experiments (*n* = 3). Different letters in the same column indicate a significant difference between the means at *p* < 0.05 in the LSD one-way ANOVA test. ^2^ mg/100 g fresh pepper fruit. ^3^ Total phenolic content (mg chlorogenic acid/g dry extract). ^4^ Total flavonoids (mg quercetin/g dry extract). ^5^ Total dihydroxycinnamic acids (mg chlorogenic acid/g dry extract). ^6^ Antiradical activity (Trolox equivalent μmol/g dry extract).

**Table 2:** Anticancer activity of ethanolic extract (E) and water (F1), methanol-water 40% (F2), and 70% (F3) fractions obtained from sweet (PS) and hot (PH) pepper fruit (IC_50_ mg/mL).

		HTC116	PC-3	L929
PS ^1^	Extract	134 ^b^ ± 4	78 ^b^ ± 4	90 ^b^ ± 3
	F1	158 ^a^ ± 4	60 ^c^ ± 3	64 ^c^ ± 3
	F2	154 ^a^ ± 5	101 ^a^ ± 3	118 ^a^ ± 3
	F3	160 ^a^ ± 4	51 ^d^ ± 4	94 ^b^ ± 3
PH	Extract	132 ^b^ ± 3	68 ^c^ ± 3	96 ^b^ ± 3
	F1	134 ^b^ ± 3	56 ^d^ ± 3	90 ^b^ ± 3
	F2	116 ^c^ ± 3	64 ^c^ ± 3	120 ^a^ ± 3
	F3	136 ^b^ ± 3	68 ^c^ ± 3	96 ^b^ ± 3
5-fluorouracil ^2^		33 ^d^ ± 3	23 ^e^ ± 3	8 ^d^ ± 1

^1^ Results for sweet pepper (PS) quoted from Chilczuk et al. 2020. The values are expressed as the mean ± SD from three independent experiments (*n* = 3). Different letters in the same column indicate a significant difference between the means at *p* < 0.05 in the LSD one-way ANOVA test. ^2^ 5-Fluorouracil (μM)—positive control.

**Table 3:** Compounds identified in sweet and hot pepper fruit extracts by UPLC-ESI-QTOF.

Retention Time	Compounds	Molecular Formula	Molecular Weight				Mass Error (ppm)	PS				PH			
			Teoretical	Observed	m/z	ion		E	F1	F2	F3	E	F1	F2	F3
0.356	Glucose (hexose)	C_6_H_12_O_6_	180.0634	180.0638	179.0565 225.0619	[M − H]^−^ [M + HCOO]^−^	2.01	+	+	+	-	+	+	+	+
0.3682	Saccharose	C_12_H_22_O_11_	342.1162	342.1166	341.1100 387.1145	[M − H]^−^ [M + HCOO]^−^	1.27	+	+	+	-	+	+	+	-
0.386	Malic acid	C_4_H_6_O_5_	134.0215	134.0221	133.0149	[M − H]^−^	4.65	+	+	-	-	+	+	+	+
0.389	Ascorbic acid	C_6_H_8_O_6_	176.0321	176.0328	175.0254 221.0335	[M − H]^−^ [M + HCOO]^−^	3.86	+	+	+	-	+	+	-	-
0.404	Quinic acid	C_7_H_12_O_6_	192.0634	192.0623	191.0549 237.0626	[M − H]^−^ [M + HCOO]^−^	0.16	+	+	-	-	+	+	+	+
0.448	Gallic acid	C_7_H_6_O_5_	170.0215	170.0223	169.0146	[M − H]^−^	4.33	+	-	+	-	-	-	-	-
0.449	Succinic acid	C_4_H_6_O_4_	118.0266	118.0269	117.0194	[M − H]^−^	2.67	+	+	-	-	+	+	-	-
0.479	Citric acid	C_6_H_8_O_7_	192.028	192.027	191.0207	[M − H]^−^	5.17	+	+	-	-	+	+	+	+
0.498	Sinapic acid -*E*-glucoside	C_17_H_22_O_10_	386.1213	386.1220	385.1148	[M − H]^−^	1.73	+	+	+	-	+	-	+	-
0.515	Ferulic acid -*E*-glucoside	C_16_H_20_O_9_	356.1107	356.1103	355.1032	[M − H]^−^	−1.31	+	-	+	-	+	-	+	-
0.565	Luteolin-6-*C*-glucoside-8-*C*-arabinoside	C_26_H_28_O_15_	580.1428	580.1436	579.1364	[M − H]^−^	1.41	+	-	-	+	+	-	-	+
0.574	Phlorentin 3′,5′-di-*C*-glucoside	C_27_H_34_O_15_	598.1198	598.1902	597.1828	[M − H]^−^	0.64	+	-	+	+	+	-	+	+
0.631	Luteolin	C_40_H_56_O_2_	286.0482	286.0487	287.0554 309.0395	[M + H]^+^ [M + Na]^+^	1.66	-	-	-	-	+	-	+	-
0.607	Quercertin 3-*O*-glucoside	C_21_H_20_O_12_	464.0955	464.0887	463.0883	[M − H]^−^	1.16	+	-	-	+	+	-	-	+
0.661	Quercetin	C_15_H_10_O_7_	302.0427	302.0423	303.0500 325.0290	[M + H]^+^ [M + Na]^+^	0.18	+	-	+	+	+	-	+	+
0.666	Quercertin 3-*O*-rhamnoside	C_21_H_20_O_11_	448.1015	448.1012	449.1084 471.0914	[M + H]^+^ [M + Na]^+^	2.04	+	-	+	+	+	-	+	+
0.672	Rutin	C_27_H_30_O_16_	610.1534	610.1545	609.1469	[M − H]^−^	1.07	+	-	+	+	-	-	-	+
0.665	Luteolin-6,8-di-*C*-glucoside	C_27_H_32_O_15_	596.1741	596.1733	597.1834	[M + H]^−^	1.07	+	+	+	+	+	-	-	-
0.689	Cinnamic acid	C_9_H_8_O_2_	148.0524	148.0519	149.0577 171.0375	[M + H]^+^ [M + Na]^+^	−2.97	+	+	-	-	+	+	-	-
0.704	Sinapic acid	C_11_H_12_O_5_	224.069	224.0685	223.0613 269.0615	[M − H]^−^ [M + HCOO]^−^	2.26	+	-	+	-	+	-	+	-
0.715	Ferulic acid	C_10_H_10_O_4_	194.0579	194.0596	193.0516 239.0604	[M − H]^−^ [M + HCOO]^−^	8.8	+	+	+	-	+	-	+	-
0.748	Luteolin-6-*C*-glucoside	C_21_H_20_O_11_	448.1006	448.101	447.0938	[M − H]^−^	1.01	+	+	+	-	+	-	+	-
0.748	Apigenin-6-*C*-glucoside	C_21_H_20_O_10_	432.156	432.1049	477.1027	[M + HCOO]^−^	−1.82		-	+	-	+	-	+	+
0.815	Blumenol-*C*-glucoside	C_19_H_32_O_7_	372.2148	372.215	417.2134	[M + HCOO]^−^	0.65	+	+	+	-	+	-	+	+
0.822	Naringenin	C_15_H_12_O_5_	272.0685	272.0679	271.0616 317.0647	[M − H]^−^ [M + HCOO]^−^	−1.93	+	-	+	+	+	-	+	+
0.829	Lioliolide	C_11_H_16_O_3_	196.1103	196.1101	197.1171 219.0985	[M + H]^+^ [M + Na]^+^	0.59	+	-	+	+	+	-	+	-
0.884	Capsianoside VII	C_38_H_64_O_17_	792,414	792.409	793.4120 815.4040	[M + H]^+^ [M + Na]^+^	−0.46	+	-	-	+	-	-	-	-
0.905	*p*-Coumaric acid	C_9_H_8_O_3_	164.0473	164.0475	163.0407 290.0452	[M − H]^−^ [M + HCOO]^−^	2.71	+	-	+	+	-	-	+	-
1.131	Naringenin-7-*O*-glucoside	C_21_H_22_O_10_	434.1213	434.1217	433.1178 479.1166	[M − H]^−^ [M + HCOO]^−^	1.04	+	+	+	+	+	-	+	-
2.421	Chlorogenic acid	C_16_H_18_O_9_	354.0945	354.0932	355.1016 377.0840	[M + H]^+^ [M + Na]^+^	−1.65	+	+	-	-	+	+	-	-
4.325	Gentisic/protocatechuic acid	C_7_H_6_O_4_	154.0266	154.0262	199.0238	[M + HCOO]^−^	−2.59	+	+	-	-	-	-	-	-
4.461	Capsianoside X	C_56_H_94_O_31_	1262.5779	1262.5786	1261.5715 1307.5587	[M − H]^−^ [M + HCOO]^−^	0.57	+	-	-	+	+	-	+	+
4.997	Capsianoside new I	C_56_H_94_O_30_	1246.5830	1246.5834	1245.5764	[M + HCOO]^−^	0.29	+	-	-	+	+	-	-	+
5.027	Capsianoside III	C_50_H_84_O_26_	1100.5251	1100.5250	1099.5180	[M − H]^−^ [M + HCOO]^−^	−0.03	+	-	-	+	+	-	-	+
5.127	Capsianoside II	C_50_H_84_O_25_	1084.5302	1084.5252	1083.5216 1129.5126	[M − H]^−^ [M + HCOO]^−^	−4,59	+	-	-	+	+	-	-	+
5.310	Capsianoside I	C_32_H_52_O_14_	660.3357	660.3359	659.3287	[M − H]^−^	0.34	+	-	+	+	+	-	+	+
5.293	Capsianoside new II	C_53_H_86_O_29_	1186.5255	1186.5252	1185.5181	[M − H]^−^	−0.2	+	-	+	+	+	-	+	+
5.576	Capsianoside IX	C_44_H_74_O_21_	938.4723	938.4667	937.4639 983.4620	[M − H]^−^ [M + HCOO]^−^	−5.93	+	-	-	+	+	-	-	+
5.809	Capsianoside new III	C_53_H_86_O_28_	1170.5306	1170.5306	1169.5239 1215.4931	[M − H]^−^ [M + HCOO]^−^	0.05	+	-	+	+	+	-	+	+
6.942	Dihydrocapsiate	C_18_H_28_O_4_	308.1988	308.1988	307.1920	[M − H]^−^	0.28	+	-	-	-	+	+	+	+
7.282	Capsianoside IV	C_32_H_52_O_13_	644.3408	644.3405	643.3337	[M − H]^−^	−0.4	+	+	+	+	-	-	-	+
7.308	Capsiate	C_18_H_26_O_4_	306.1831	306.1831	305.1756	[M + HCOO]^−^	−0.53	+	-	-	-	-	-	-	-
7.591	Capsianoside C	C_82_H_134_O_38_	1726.8553	1726.8538	1725.8467	[M − H]^−^	−0.9	+	-	-	-	+	-	-	+
7.807	Capsaicin	C_18_H_27_NO_3_	305.1991	305.1996	304.1924 350.1979	[M − H]^−^ [M + HCOO]^−^	1.56	-	-	-	-	+	-	-	+
8.207	Nordihydrocapsiate	C_17_H_26_O_4_	274.1831	294.1832	293.1759 339.1779	[M − H]^−^ [M + HCOO]^−^	0.19	+	-	-	-	+	+	+	+
8.423	Capsianoside F	C_82_H_134_O_37_	1710.8604	1710.8618	1709.8543	[M − H]^−^	0.8	+	+	+	+	+	-	-	+
8.690	Dihydrocapsaicin	C_18_H_29_NO_3_	307.2147	307.2151	306.2079 352.2133	[M − H]^−^ [M + HCOO]^−^	1.22	-	-	-	-	+	-	-	+
8.988	Capsianoside I methyl	C_33_H_54_O_14_		674.3488	675.3561	[M + H]^+^	−3.86	-	-	-	-	+	-	-	-
10.504	Oxylipin	C_18_H_32_O_3_	296.2351	296.2354	295.2281	[M − H]^−^	0.71	+	-	-	+	+	+	+	+
10.618	Methyl cinnamate	C_10_H_10_O_2_	162.0681	162.068	163.0751	[M + H]^+^	−0.77	+	+	+	+	-	-	-	-
13.342	Capsidiol	C_15_H_24_O_2_	236.1776	236.1766	295.1908	[M + HCOO]^−^	−4.22	+	+	-	-	-	-	-	-
15.433	*α*-Tocotrienol	C_29_H_44_O_2_	424.3341	424.3348	423.3276 469.3328	[M − H]^−^ [M + HCOO]^−^	1.63	+	+	+	-	+	-	+	+
16.898	β-Tocotrienol	C_28_H_42_O_2_	410.3185	410.3191	409.3118	[M − H]^−^	1.45	+	+	+	+	+	+	+	+

## Data Availability

Not applicable.

## Sample Availability

Samples of the compounds are not available from the authors.
